# Supplementary material for: Development of explicit definitions of potentially inappropriate prescriptions for antidiabetic drugs in patients with type 2 diabetes: A multidisciplinary qualitative study
Source: PLoS One. 2024 Sep 27;19(9):e0309290. doi: 10.1371/journal.pone.0309290 (PMC11432865; doi:10.1371/journal.pone.0309290)
Supplement: S1 Table — (PDF) [file pone.0309290.s001.pdf]

Supplementary information SI1: COREQ checklist

No Item	Guide questions/description	Location where item is reported
**Domain 1: Research team and reflexivity**		
Personal Characteristics		
**1. Interviewer/facilitator**	Which author/s conducted the interview or focus group?	Page 5; line: 90
**2. Credentials**	What were the researcher's credentials? E.g. PhD, MD	Page 5; line: 90
**3. Occupation**	What was their occupation at the time of the study?	Page 5; line: 90
**4. Gender**	Was the researcher male or female?	Page 5; line: 90
**5. Experience and training**	What experience or training did the researcher have? Relationship with participants	Page 5; line: 84
**6. Relationship established**	Was a relationship established prior to study commencement?	Page 5; line: 84
**7. Participant knowledge of the interviewer**	What did the participants know about the researcher? e.g. personal goals, reasons for doing the research	Page 5; line: 84
**8.Interviewer characteristics**	What characteristics were reported about the interviewer/facilitator? e.g. Bias, assumptions, reasons and interests in the research topic	Page 1; line: 1
**Domain 2: study design**		
Theoretical framework		
**9.Methodological orientation and Theory**	What methodological orientation was stated to underpin the study? e.g. grounded theory, discourse analysis, ethnography, phenomenology, content analysis	Page 5; lines 91 to 98
Participant selection		
**10. Sampling**	How were participants selected? e.g. purposive, convenience, consecutive, snowball	Page 5; lines 86
**11. Method of approach**	How were participants approached? e.g. face-to-face, telephone, mail, email	Page 5; lines 86
**12. Sample size**	How many participants were in the study?	Page 8; line 135
**13. Non-participation**	How many people refused to participate or dropped out? Reasons? Setting	N/A
**14.Setting of data collection**	Where was the data collected? e.g. home, clinic, workplace	Page 5; line 89
**15. Presence of non-participants**	Was anyone else present besides the participants and researchers?	Page 5; line 90 -91
**16. Description of sample**	What are the important characteristics of the sample? e.g. demographic data, date Data collection	Page 8; line 140
**17. Interview guide**	Were questions, prompts, guides provided by the authors? Was it pilot tested?	Page 5; line 91 -92
**18. Repeat interviews**	Were repeat interviews carried out? If yes, how many?	N/A
**19. Audio/visual recording**	Did the research use audio or visual recording to collect the data?	N/A
**20. Field notes**	Were field notes made during and/or after the interview or focus group?	Page 6; line 95
**21. Duration**	What was the duration of the interviews or focus group?	Page 5; lune 90
**22. Data saturation**	Was data saturation discussed?	Page 5; lune 94
**23. Transcripts returned**	Were transcripts returned to participants for comment and/or correction?	Page 7; line 123
**Domain 3: analysis and findings**		
Data analysis		
**24. Number of data coders**	How many data coders coded the data?	Page 6; line: 102
**25.Description of the coding tree**	Did authors provide a description of the coding tree?	N/A

**26. Derivation of themes**	Were themes identified in advance or derived from the data?	N/A
**27. Software**	What software, if applicable, was used to manage the data?	Page 6; line 95
**28. Participant checking**	Did participants provide feedback on the findings? Reporting	Page 11; line 174
Reporting		
**29. Quotations presented**	Were participant quotations presented to illustrate the themes / findings? Was each quotation identified? e.g. participant number	N/A
**30. Data and findings consistent**	Was there consistency between the data presented and the findings?	Page 14; line 190
**31. Clarity of major themes**	Were major themes clearly presented in the findings?	Page 14; line 190
**32. Clarity of minor themes**	Is there a description of diverse cases or discussion of minor themes?	Page 14; line 190
