# Supplementary material for: Development of explicit definitions of potentially inappropriate prescriptions for antidiabetic drugs in patients with type 2 diabetes: A multidisciplinary qualitative study
Source: PLoS One. 2024 Sep 27;19(9):e0309290. doi: 10.1371/journal.pone.0309290 (PMC11432865; doi:10.1371/journal.pone.0309290)
Supplement: Attachment — Submitted filename: Response to Reviewers.docx [file pone.0309290.s002.docx]

**Response to Journal Requirements:**

We thank Academic Editor for comments and constructive suggestions. We have carefully revised the manuscript according to each comment and suggestion. In the following, our responses are given in blue.

1. Please ensure that your manuscript meets PLOS ONE's style requirements, including those for file naming. The PLOS ONE style templates can be found at 
<https://journals.plos.org/plosone/s/file?id=wjVg/PLOSOne_formatting_sample_main_body.pdf> and 
<https://journals.plos.org/plosone/s/file?id=ba62/PLOSOne_formatting_sample_title_authors_affiliations.pdf>

Thank you for your comment. We have taken the necessary steps to correct the elements that were not correctly entered in accordance with PLOS ONE's style requirements.

**Modifications: In the title page, we have removed the physical address of the corresponding author and specified his initials line 14 of the revised manuscript with track changes.**

**According to the PLOS ONE style templates,**

- **We have used a font size of 18pt font for all level 1 headings and 16pt fond for level 2 headings.**
- **We have changed the style of references by using square brackets instead of parentheses.**
- **We have deleted lines 17 to 19 of the revised manuscript with track changes specifying keywords and competing interests.**
- **On line 207 of the revised manuscript with track changes, we have rewritten fig 1 instead of figure 1.**
- **In the online submission system, we have changed the file "Figure_1.tiff" to "Fig1.tiff" and the file "Supplementary_information_S1" to S1 Table.pdf".**

2. Thank you for including your ethics statement:  "Participants were informed of the study purpose, questions, and procedure prior to the nominal groups. Verbal consent was obtained at the beginning of both nominal groups because the study presented no more than minimal risk of harm to subjects and involved no procedures for which written consent is normally required outside of the research context.".

Please amend your current ethics statement to include the full name of the ethics comité/institutional review board(s) that approved your specific study.

Once you have amended this/these statement(s) in the Methods section of the manuscript, please add the same text to the “Ethics Statement” field of the submission form (via “Edit Submission”).

For additional information about PLOS ONE ethical requirements for human subjects research, please refer to <http://journals.plos.org/plosone/s/submission-guidelines#loc-human-subjects-research>.

**Modifications: We have amended our current ethics statement to include the full name of the ethics committee, and we have amended this statement in the Methods section of the manuscript, line 158 in the revised manuscript with track changes "This study was approved by the University of Lille Research Ethics Committee"; and we have added the same text to the "Ethics Statement" field of the submission form (via "Edit Submission").**

3. We noticed a mistake in your MS regarding recruitment dates. Please ensure this is corrected.

Thank you for spotting this mistake. We have corrected it, line of the revised manuscript with track changes.

**Modification: In line 150 of the revised manuscript with track changes, we have changed " 2022 " instead of " 2024 ".**

4. We note that the grant information you provided in the ‘Funding Information’ and ‘Financial Disclosure’ sections do not match.

When you resubmit, please ensure that you provide the correct grant numbers for the awards you received for your study in the ‘Funding Information’ section.

We have corrected the indicated items. We apologize for any errors in translation.

This research was funded by PreciDIAB, which is jointly supported by the French National Agency for Research (ANR- 18- IBHU- 0001), by the European Union (FEDER - agreement NP0025517), by the Hauts- de- France Regional Council (agreement 20001891/NP0025517) and by the European Metropolis of Lille (MEL, agreement 2019_ESR_11).

The funder had no role in study design, data collection and analysis, decision to publish, or preparation of the manuscript.

**Response to Reviewer 1 Comments**

We thank Reviewer 1 for comments and constructive suggestions. We have carefully revised the manuscript according to each comment and suggestion. In the following, our responses are given in blue.

**Point 1.** Title- few readers will know what a nominal group approach is…I would reserve this to the abstract and to the text where this team can be defined.

Thank you for your comment. We have changed this part of the title to read: "a multidisciplinary qualitative study".

**Modification (title): Development of explicit definitions of potentially inappropriate prescriptions for antidiabetic drugs in patients with type 2 diabetes: a multidisciplinary qualitative study.**

**Point 2.** Cover page- who funded the project? The cover indicates that there is no conflict of interest.

Project funding has been declared on the plosOne submission platform. The authors declare that they have no financial or non-financial conflicts of interest.

This project is part of the PreciDIAB project of the National Center for Precision Diabetic Medicine, which brings together several teacher-researchers and practitioners from the University of Lille and the Lille University Hospital. More information: <https://www.precidiab.org/en/>

The funder had no role in study design, data collection and analysis, decision to publish, or preparation of the manuscript.

**Modifications: We deleted line 19 of the revised manuscript with track changes specifying competing interests according to the PLOS ONE style templates (see responses to Editor’s comments).**

**Point 3.** Abstract - Purpose—has the management significantly changed or has there been an introduction of numerous new drugs and if so—could you provide an estimate of how large “significant changes” are with respect to number of new drugs for T2DM?

Thank you to the reviewer for this comment. The word "significant" was not correct. As shown by the work of Chong et al. (2023) [https://doi.org/10.1002/kjm2.12800] and Galindo et al. (2022) [https://doi.org/10.1136/ bmjmed-2022-000372], the management of patients living with type 2 diabetes has evolved over the last 10 years with new molecules, in particular SGLT-2 inhibitors, GLP-RAs and DPP4 inhibitors, in the choice of first-line treatments and combinations of therapies.

**Modifications: In line 27 in the revised manuscript with track changes, we deleted the word “significant”. As a result, we have also removed the word “significant” from the introductory section, line 74.**

**Point 4.** Abstract - How does one know that screening tools could limit the numbers of potentially inappropriate drugs? Could the screening tools make matters more confusing—is there precedent for screening tools to improve rather than harm physician ordering (i.e by creating confusion and costing time)?

Explicit criteria for potentially inappropriate prescribing (PIP) in older people have gained attention since the first publication of the Beers criteria in 1991. Explicit criteria are generally used as rigid standards and do not take into account individual differences among patients, nor the complexity and appropriateness of entire medication regimens [https://doi.org/10.1001/archinte.1991.00400090107019]. The effectiveness of explicit tools such as lists of potentially inappropriate prescriptions has been largely demonstrated. Several tools were used to assess PIM in hospitalized patients over 65most commonly Beer's criteria and the STOPP/START tool. In a systematic review of the literature, Alshammari et al. showed a reduction of PIM ranged from 3.5% up to 87% [https://doi.org/10.2147/DHPS.S303101].

We agree with the reviewer that for 15 years, screening tools implemented in clinical decision support systems (CDSS) have caused harm to physician ordering. This was due to several factors, including data quality and human factors [http://dx.doi.org/10.1016/j.jbi.2015.03.006]. However, CDSS have improved over the last decade and their effectiveness has been demonstrated in recent large RCTs [https://doi.org/10.1016/S0140-6736(23)02465-0 and https://doi.org/10.1001/jama.2024.6259].

**Modifications: Lines 31-32 in the revised manuscript with track changes, the effectiveness of explicit tools such as lists of potentially inappropriate prescriptions has been widely demonstrated.**

**Point 5.** Abstract - Methods: COREG should be in the paper, not necessarily in the abstract. Nominal group should be defined in the methods section of the abstract.

We agree with the reviewers. We have corrected the sentence.

**Modifications:**

**Lines 39-40 in the revised manuscript with track changes, the sentence mentioning COREQ criteria has been removed from the abstract.**

**Lines 126-130 in the revised manuscript with track changes, the COREQ criteria has been defined in the study design paragraph.**

**Lines 37-38 in the revised manuscript with track changes, the nominal group has been defined in the methods paragraph of the abstract.**

**Point 6.** Abstract - Results: is there any information on validity and reliability of the definitions. Did the steering committee vote on each definition?

This work focused on the feasibility of generating an explicit list of criteria, and the steering committee and nominal groups validated the explicit nature of the proposed definitions. The nominal group technique can generate new proposals, but consensus on the validity of definitions and their use in practice remains limited, as the nominal group technique requires small groups of participants. Baclet et al. (2022) [https://doi.org/10.1016/j.idnow.2022.02.004] set up a qualitative study to Explicit definitions of potentially inappropriate prescriptions of antibiotics in hospitalized older patients and validated this list using a delphi survey in 2024 [https://doi.org/10.3390/ antibiotics13030283].

Consequently, this study did not provide results on the validity or reliability of the definitions. The validity of these definitions will be investigated on a larger panel of participants, using a consensus method such as the Delphi method.

**Point 7.** Abstract - Conclusion: this is very overstated- the conclusion should be that this is a great pilot effort- and should be redone in more settings in France for validation and potential pilot data. Is a Delphi panel the next step or is a program that included more nominal groups needed?

Thank you for your suggestions regarding the conclusion of the abstract. As described in our protocol, obtaining and validating this list of definition is a 3-step process [https://doi.org/10.3390/healthcare9111539]. The Delphi method will be the next step in this study, as the delphi survey is an appropriate way to reach consensus and allows for the involvement of many participants. We modified the conclusion of the abstract in the revised version of the manuscript.

**Modifications:**

**Lines 49-52 in the revised manuscript with track changes, this qualitative study develop a specific list of explicit definitions of potentially inappropriate prescriptions of antidiabetic drugs in patients with type 2 diabetes mellitus by gathering the opinions of healthcare professionals caring for patients with type 2 diabetes.** **Although the new list provides valuable insights, it will be validated by expert consensus in a Delphi survey before being implemented in practice.**

**Point 8.** Abbreviations- there are too many here…. Please try to decrease the number of abbreviations.

Thanks for your comment, we've reduced the number of abbreviations used.

**Modifications: We have removed the 6 following abbreviations:**

**ADA: American Diabetes Association, ATC: Anatomical Therapeutic Chemical, EASD: European Association for the Study of Diabetes, SFD: Société francophone du diabète, SmPC: summary of product characteristics and CDSS: clinical decision support system**

**We now use only 7 abbreviations instead of 13:**

- **AD: antidiabetic**
- **DDP-4: dipeptidyl-peptidase-4**
- **eGFR: estimated glomerular filtration rate**
- **GLP-1 RA: inhibitors, glucagon-like peptide 1 receptor agonist**
- **PIP: potentially inappropriate prescription**
- **SGLT2: sodium-glucose cotransporter-2**
- **T2DM: type 2 diabetes mellitus**

**Point 9.** Introduction- again—too many abbreviations.

Thanks for your comment.

**Modifications: we've reduced the number of abbreviations used in the text.**

**Point 10.** The description of implicit and explicit needs more context and references. Many studies formally compare explicit and implicit evaluations and should be cited…These studies discuss pros and cons of each method.

We thank the reviewer for this comment. In clinical pharmacology, two approaches can be used to assess inappropriateness of prescribing: the first is based on so-called 'implicit' expert judgement, and the second uses explicit criteria. An implicit judgement is based on an expert's assessment taking into account research data, clinical circumstances and patient preferences [https://doi.org/10.1007/s40266-018-0554-2]. In contrast, explicit criteria are based on predefined rules for analysing drug prescriptions and do not require the intervention of an expert.

Implicit approach can use standardized tools, such as Medication Appropriateness Index (MAI) [https://doi.org/10.1007/s40266-013-0118-4]. This tool requires the experts to follow several steps, including (…). MAI has demonstrated relevant (Hanlon), but RCT are still missing for implicit approaches (https://doi.org/10.1007/s40266-018-0554-2). Explicit approaches, on the other hand, can use various lists or CDSS tools. The effectiveness of explicit tools such as lists of potentially inappropriate prescriptions has been well documented [https://doi.org/10.2147/DHPS.S303101. Several tools were used to assess PIM in hospitalized patients over 65most commonly Beer's criteria and the STOPP/START tool. A systematic review of the literature by Alshammari et al. reported reductions in PIM ranging from 3.5% to 87% [https://doi.org/10.2147/DHPS.S303101]. We also included references to the work of Hill-Taylor et al. [https://doi.org/10.1111/jcpt.12059], Lopez-Rodriguez et al. [https://doi.org/10.1371/journal.pone.0237186].

Explicit and implicit approaches are considered complementary. Lopez-Rodriguez [https://doi.org/10.1371/journal.pone.0237186] showed that explicit criteria are easier to implement and easier to automate. The REMEDIES list proposed a mixed approach, combining implicit and explicit recommendations. Lastly, the IGRIMUP recommended to combine these approaches [https://doi.org/10.1007/s40266-018-0554-2].

In line with the reviewer’s comment, we added some sentences to give more context and references for implicit and explicit approaches in the introduction section :

**Modifications:**

**Lines 88-99 in the revised manuscript with track changes, we have added the parts in bold in the following text:**

**The MAI is the most widely accepted implicit method.** **However, the implicit approach has practical limitations, as itis time-consuming and requires specialized knowledge and skills to be applied.**

It is also possible to reduced PIPs by applying an explicit approach, i.e. explicit criteria that combine the various items of information related to the prescription in the absence of an expert assessment **using predefined criteria** (e.g. a prescription of metformin in a patient with an estimated glomerular filtration rate (eGFR) below 30 mL/min/1.73 m² is a PIP). **The effectiveness** **of this explicit approach in detecting PIPs has been well demonstrated**.

**These two approaches, implicit and explicit, should be combined for optimal patient management.** With the increasing use of computerized physician order entry and electronic medical records, the explicit approach becomes more practical and easier to implement compared to integrating implicit criteria**.**

**Point 11.** Methods - The context for study design needs more descriptions as well--- in the introduction or methods. More text on when to use COREQ is also needed. Is there background about how many persons should be in the nominal groups and how many should be on the steering committee. Are there previous studies that have used the nominal group methods—what did they do and what did they find?

Thanks for your comment, we've clarified the context of our study design at the end of the introduction.

In the method section, we have also included a reference to the use of the COREQ checklist throughout the study. The number of people included in a nominal group varies according to the reference, but it must be a manageable group size. Allen et al. [https://doi.org/10.12968/bjcn.2004.9.3.12432] recommended between nine and twelve participants. McMillian et al. [https://doi.org/10.1007/s11096-016-0257-x] suggested between two and fourteen participants.

Baclet et al. (2022) [https://doi.org/10.1016/j.idnow.2022.02.004] set up a qualitative study to define explicit definitions of potentially inappropriate prescriptions of antibiotics in hospitalized older patients.

Beuscart et al. (2018) [https://doi.org/10.1186/s12916-018-1007-9] set up a qualitative study to define international core outcome set for clinical trials of medication review in multi-morbid older patients with polypharmacy.

The nominal group technique is used in healthcare to survey professionals and determine new outcomes. For example, The 2023 ACR/EULAR defined Antiphospholipid Syndrome Classification Criteria using this method [https///doi.org/10.1002/art.42624], Michel et al. employed an online nominal group technique to identify key implementation factors for COVID-19 vaccination programs in community pharmacies [https://doi.org/[10.1007/s11096-021-01335-x](https://doi.org/10.1007/s11096-021-01335-x) ], Romera-liebana et al. used the nominal group technique to assess frailty in the person with type 2 diabetes mellitus [https://doi.org/10.1016/j.rceng.2023.09.008].

**Modifications :**

**Line 119-121 in the revised manuscript with track changes**, **we specify that this work involves using a qualitative approach, an approach that has already been used to set up explicit definitions of potentially inappropriate prescriptions of antibiotics in hospitalized older patients.**

**Line 147-148 in the revised manuscript with track changes, […] following the recommendations of Allen et al. and McMillian et al.**

**Point 12.** Why move directly to explicit—why not do implicit and explicit and then compare findings?

The overall aim of our research is to propose new criteria for implementation in clinical decision support systems (CDSS). Implementing rules in a CDSS requires explicit criteria as show by Robert et al. [https://doi.org/ 10.1111/bcp.15898], Crowley et al. [https://doi.org/10.1186/s12913-020-5056-3] or Tsopra et al. [https://doi.org/ 10.1093/jamia/ocz057] Therefore, we focused on developing explicit criteria. We have previously published our study protocol to achieve this aim. Additionally, implicit guidelines already exist and are mainly found in the guidelines of learned societies.

Explicit and implicit approaches are considered complementary. Lopez-Rodriguez [https://doi.org/ 10.1371/journal.pone.0237186] showed that explicit criteria are easier to implement and easier to automate. The REMEDIES list proposed a mixed approach, combining implicit and explicit recommendations. Lastly, the IGRIMUP recommended combining these approaches [https://doi.org/10.1007/s40266-018-0554-2]. In a previous paper, our research team showed that using CDSS by clinical pharmacists improved antidiabetic drug management in hospitalized patients with T2DM [https://doi.org/ 10.1111/bcp.15898]. Most of the pharmaceutical interventions suggested by the CDSS were not flagged by clinical pharmacists using the implicit approach. This study shows that additional methods beyond those proposed in this manuscript are needed to compare implicit and explicit criteria.

**Point 13**. Recruitment - why 5 to 15 persons per group? How random was the selection process? Is the process of selection biased- and will this affect the results? How was the group makeup determined? Was there any training? Had the facilitators prior experience with nominal groups? Has the team succeeded in nominal group explicit checklist development?

The number of people included in a nominal group varies according to the reference, but it must be a manageable group size. Allen et al. (2004) [https://doi.org/10.12968/bjcn.2004.9.3.12432] recommended between 9 and 12 participants. McMillian et al. (2016) [https://doi.org/10.1007/s11096-016-0257-x] suggested between two and fourteen participants.

Baclet et al. (2022) [https://doi.org/10.1016/j.idnow.2022.02.004] used a similar approach with 28 participants to define explicit definitions of potentially inappropriate prescriptions of antibiotics in hospitalized older patients.

As discussed by Allen et al (2004) [https://doi.org/10.12968/bjcn.2004.9.3.12432]. and Olsen et al. (2019) [https://doi.org/10.1177/1609406919866049], due to the commitment required by the participants, a truly random sample may not be appropriate. Therefore, people who are recruited opportunistically, but who can answer the key research questions, may be a more reliable group.

The selection of the various groups and participants was therefore made in conjunction with the steering committee to focus on the healthcare professionals to be in charge of prescribing and dispensing antidiabetics. The decisions were taken with the members of the steering committee, some of whom had already carried out qualitative studies such as Beuscart et al. [https://doi.org/10.1186/s12916-018-1007-9], Calafiore et al. [https://doi.org/ 10.3399/BJGP.2021.0626].

The two investigators present during the nominal groups (EG and PQ) were trained in this technique by Dr. Michel CUNIN, expert in qualitative research at the Department of General Practice, Faculty of Medicine, Lille.

**Point 14.** What is an explicit vs implicit definition?

In clinical pharmacology, two approaches can be used to assess inappropriateness of prescribing: the first is based on so-called 'implicit' expert judgement, and the second uses explicit criteria. An implicit judgement is based on an expert's assessment taking into account research data, clinical circumstances and patient preferences (see D&A). In contrast, explicit criteria are based on predefined rules for analyzing drug prescriptions and do not require expert intervention.

Implicit approach can use standardized tools, such as Medication Appropriateness Index (MAI) [https://doi.org/10.1007/s40266-013-0118-4]. This tool requires the experts to follow several steps, including (…). MAI has demonstred relevant (Hanlon), but RCT are still missing for implicit approaches (https://doi.org/10.1007/s40266-018-0554-2). Explicit approaches, on the other hand, can use variouse lists or CDSS tools. The effectiveness of explicit tools such as lists of potentially inappropriate prescriptions has been well documented [https://doi.org/10.2147/DHPS.S303101. Several tools were used to assess PIM in hospitalized patients over 65most commonly Beer's criteria and the STOPP/START tool. A systematic review of the literature by Alshammari et al. reported reductions in PIM ranging from 3.5% to 87% [https://doi.org/10.2147/DHPS.S303101]. We also included references to the work of Hill-Taylor et al. [https://doi.org/10.1111/jcpt.12059], Lopez-Rodriguez et al. [https://doi.org/ 10.1371/journal.pone.0237186].In line with the reviewer’s comment, we have now added more precision in the revised version of our manuscript.

**Modifications:**

**Lines 135-145 in the revised manuscript with track changes:**

**3.2.1	Explicit definitions**

**The inappropriateness of drug prescriptions can be evaluated in clinical pharmacology using two approaches: the first is based on a so-called “implicit” expert judgement, and the second uses explicit criteria. An implicit judgement is based on an expert’s evaluation of the quality of care with regard to the patient’s situation and guidelines on the use of antimicrobial drugs. In contrast, explicit criteria are based on predefined rules for the analysis of drug prescriptions, and do not require intervention by an expert.**

**3.2.2	Potentially inappropriate prescription**

**Explicit definitions cover situations considered by experts to be generally inappropriate, as defined in the literature or by expert consensus. However, when an explicit definition is applied to a given prescription, the absence of expert opinion means that the prescription inappropriateness cannot be confirmed. Therefore, explicit definitions correspond to potentially inappropriate prescriptions.**

**Point 15.** Results

It would be preferable to included four columns in the table—one for each of the three groups and then the overall characteristics (that are shown)

**Thank you for your comment. We have added the proposed elements to the table.**

**Modifications: lines 204 in the revised manuscript with track changes.**

| Characteristics | Participants n=30 (100.0%) | General practitioners  n = 6 (20.0%) | Diabetologists  n = 10 (33.3%) | Pharmacists  n = 14 (46.6%) |
| --- | --- | --- | --- | --- |
| Age (median (range)) | 42.5 (27; 67) | 48.5 (40; 67) | 47 (29; 59) | 39.5 (27; 59) |
| Years since qualification (MD or PharmD, median (range)) | 15 (2; 41) | 22 (9; 40) | 20 (1; 31) | 9 (1; 37) |
| Females | 17 (56.6%) | 0 (0.0%) | 6 (60%) | 11 (78.6%) |
| Involved in antidiabetic drug stewardship | 15 (50.0%) | 5 (83.3%) | 8 (80.0%) | 2 (14.3%) |

**Point 16.** Discussion - Findings—does the nominal literature state that a list that is generated needs to be externally validated? Why not compare explicit versus implicit now? This is a nice start, but several limitations are noted—some by the authors themselves? I would have preferred to see possibly a second independent effort done and then compare the final results of each effort. This reads more like a QI effort than a research study.

We thank the reviewer for this comment. The study and results presented in our article are part of a research protocol based on previous published research on inappropriate prescribing and research approaches recommended for the development of core outcome set [https://doi.org/10.3390/healthcare9111539; https://doi.org/10.3390/antibiotics13030283; https://doi.org/10.1186/s12916-018-1007-9]. We chose to carry out this project in three stages: (i) Identifying existing knowledge, (ii) eliciting opinions with stakeholder involvement, and (iii) to developing consensus methods. This approach was used by other researchers to develop new explicit tools, such as Roux et al. who create the REMEDI[e]S tool (REview of potentially inappropriate MEDIcation pr[e]scribing in Seniors) [https://doi.org/10.1007/s00228-021-03145-6]. Our work is based on validated methods already used in this field of research. Baclet et al. (2022) [https://doi.org/10.1016/j.idnow.2022.02.004] set up a qualitative study to define explicit definitions of potentially inappropriate prescriptions of antibiotics in hospitalized older patients. As Williamson's work on core outcome sets shows, qualitative methods are useful for creating knowledge [https://doi.org/10.1186/1745-6215-13-132]. Consequently, the work presented in our manuscript should not be considered merely as qualitative improvement but as rigorous research work.

First, we conducted a systematic review of the literature, published in PlosOne [https://doi.or g/10.1371/journal.pone.0274256]. Our results showed that researchers primarily focused on at-risk situations related to biguanide prescriptions in patients with renal dysfunction and sulfonylureas prescription in older people. The systematic review revealed a lack of consensus on explicit definitions, which were heterogeneous and generally limited to a small number of drugs and clinical situations.

Then, we conducted this qualitative study to extract new insights from the systematic review. The nominal group technique is used in healthcare to survey professionals and determine new outcomes. For example, The 2023 ACR/EULAR for defining Antiphospholipid Syndrome Classification Criteria [https///doi.org/10.1002/art.42624], Michel et al. using an online nominal group technique to identifiy key implementation factors for COVID-19 vaccination programs in community pharmacies [https://doi.org/[10.1007/s11096-021-01335-x](https://doi.org/10.1007/s11096-021-01335-x) ], Romera-liebana et al. employed the nominal group technique to assess frailty in the person with type 2 diabetes mellitus [https://doi.org/10.1016/j.rceng.2023.09.008].

Using this approach, we identified explicit definitions that were not found in the systematic review. We have already performed the so-called “consultation meeting” (see Williamson et al. and example in Baclet et al. or Beuscart et al.) This is a complex process in which definitions from the systematic review and the qualitative studies are compared, re-formulated, and pooled. We applied a rigorous methodology and found that 24 definitions from our qualitative study were completely new and had not been reported in previous published studies (based on our systematic review). Additionally, 10 definitions were more detailed than those found in our systematic literature review. Specifically, we identified and classified the validated explicit definitions in the following contexts: (i) the potential need to temporarily discontinue a medication in the event of acute illness, (ii) the potential need to adjust the dosage regimen , (iii) inappropriate treatment initiation, and (iv) the need for additional monitoring in the management of a patient with T2DM, with more precision than on the potentially inappropriate nature of the criteria. For example, the need to suspend treatment in an acute situation or the need to re-evaluate and optimize dosing in a chronic situation such as acute or chronic liver disease.

We did not present these results as they require expanded methods, results, and discussion, which were not directly requested by the reviewer’s comment. However, we could include these findings in a revised version of the manuscript if the reviewer believes that doing so would enhance the relevance of our manuscript for the readers of PlosOne.

Conversely, comparing implicit and explicit approaches was beyond the scope of our study. Furthermore, various studies and authors suggest that both approaches are complementary and should be combined rather than opposed. [https://doi.org/10.1007/s00228-019-02634-z].

**Response to Reviewer 2 Comments**

We thank Reviewer 2 for comments and constructive suggestions. We have carefully revised the manuscript according to each comment and suggestion. In the following, our responses are given in blue.

**Point 1.** In the introduction, add more findings to discuss the significance of your study, especially why non-specialist physicians would prescribe AD prescriptions for T2DM patients and why it’s an issue in your country and globally?

Thank you for your comment. We agree with the reviewer that this issue is not specific to France. The management of patients with type 2 diabetes by general practitioners has been pointed out in several studies and several issue have been raised in studies in the USA [https://doi.org/10.1186/1471-2458-9-367], Norway [https://doi.org/10.1111/dme.14586], Ireland [<https://doi.org/10.1016/j.pcd.2009.09.002>], Australia [https://doi.org/10.1186/s12875-020-1102-9] and Cameroon [https://doi.org/10.1186/s12902-015-0016-3]. In France, the results of the ENTRED study (published in PlosOne) shows that 90% of patients living with type 2 diabetes are followed up by their general practitioners.

**Modifications:**

**Lines 80-82: in the revised manuscript with track changes,**

**Medical management of patients with diabetes has been the subject of recently updated professional consensus and clinical practice guidelines. Patients with type 2 diabetes are mainly managed by general practitioners and several studies showed that guidelines are insufficiently applied among patients followed by general practitioners.**

**Point 2.** In the introduction, discuss the most updated version of ADA guidelines to treat T2DM patients, and the limitations of the implicit approach to T2DM treatment.

We thank the reviewer.

As shown by the work of Chong et al. (2023) [https://doi.org/10.1002/kjm2.12800] and Galindo et al. (2022) [https://doi.org/10.1136/ bmjmed-2022-000372], the management of patients living with type 2 diabetes has evolved over the last 10 years with new molecules, in particular SGLT-2 inhibitors, GLP-RAs and DPP4 inhibitors, in the choice of first-line treatments and combinations of therapies not just metformin.

We have modified the manuscript. We have also included citations to the work of Hill-Taylor et al., Lopez-Rodriguez et al. and Hanlon et al. showing the strengths and weaknesses of implicit and explicit approaches. The two major limitations of the implicit approach are: (i) the need of an available expert to assess drug prescriptions and (ii) time-consuming procedures to perform adequately an implicit evaluation. Experts able to perform implicit evaluation of the antidiabetic prescriptions are missing or are overload and do not have enough time to perform implicit medication review among all diabetic patients. Conversely, the number of diabetic patients is growing.

**Modifications:**

**Lines 73-75: Over the last decade, the management of patients with type 2 diabetes mellitus (T2DM) has undergone significant changes, with new molecules, in particular SGLT-2 inhibitors, GLP-1 receptor agonists and DPP-4 inhibitors, in the choice of first-line treatments and combinations of therapies.**

**Lines 80-82: in the revised manuscript with track changes,**

**Medical management of patients with diabetes has been the subject of recently updated professional consensus and clinical practice guidelines. Patients with type 2 diabetes are mainly managed by general practitioners and several studies showed that guidelines are insufficiently applied among patients followed by general practitioners.**

**Lines 88-99 in the revised manuscript with track changes, we have added the parts in bold in the following text:**

**The MAI is the most widely accepted implicit method. However, the implicit approach has practical limitations, as itis time-consuming and requires specialized knowledge and skills to be applied.**

It is also possible to reduced PIPs by applying an explicit approach, i.e. explicit criteria that combine the various items of information related to the prescription in the absence of an expert assessment using **predefined criteria** (e.g. a prescription of metformin in a patient with an estimated glomerular filtration rate (eGFR) below 30 mL/min/1.73 m² is a PIP). **The effectiveness of this explicit approach in detecting PIPs has been well demonstrated**.

**These two approaches, implicit and explicit, should be combined for optimal patient management.** With the increasing use of computerized physician order entry and electronic medical records, the explicit approach becomes more practical and easier to implement compared to integrating implicit criteria**.**

**Point 3.** The conclusion needs revision to highlight your important findings.

Thank you for your comment. Line 299-303, we have modified our conclusion by specifying that our qualitative study involved 30 healthcare professionals and generated 38 explicit definitions of PIP-Ads classified into 4 contexts: (i) the potential need to temporarily discontinue a medication in the event of acute illness, (ii) the potential need to adjust the dosage regimen, (iii) inappropriate treatment initiation, and (iv) the need for additional monitoring in the management of a patient with T2DM.
