# Supplementary material for: Development of explicit definitions of potentially inappropriate prescriptions for antidiabetic drugs in patients with type 2 diabetes: A multidisciplinary qualitative study
Source: PLoS One. 2024 Sep 27;19(9):e0309290. doi: 10.1371/journal.pone.0309290 (PMC11432865; doi:10.1371/journal.pone.0309290)
Supplement: Attachment — Submitted filename: Response to Reviewers.docx [file pone.0309290.s003.docx]

**Response to Journal Requirements:**

We thank Academic Editor for comments and constructive suggestions. We have carefully revised the manuscript according to each comment and suggestion. In the following, our responses are given in blue.

**Dear authors**

I propose that both the abstract conclusion and the text conclusion explicitly state the necessity of validating the 38 explicit definitions by healthcare experts from various settings across France before proceeding to the Delphi survey.

On line 287 of the manuscript conclusion it is stated "The most relevant and useful definitions for clinical practice must now be selected by expert consensus to produce a tool that can help non-diabetologists prescribe drugs". The requirement to pick "Must now" should be eliminated, as this qualitative study solely proposes explicit concepts that necessitate additional confirmation.

We thank the editor for this comment. The validation of the 38 explicit definitions is currently ongoing through a Delphi survey among healthcare experts from various settings across France. This survey is indeed providing additional confirmation and content to the definitions (e.g. rephrasing, additional details, vote). In line with the Editor’s comment, we changed the conclusion of the abstract and in the conclusion of the article.

**Modifications in line 46 of the revised manuscript with track changes: “***This list of 38 explicit definitions necessitates additional confirmation by expert consensus before use in clinical practice***.”**

**Modifications in line 328 (previous line 287) of the revised manuscript with track changes:**

- We removed the sentence: *“The most relevant and useful definitions for clinical practice must now be selected by expert consensus to produce a tool that can help non-diabetologists prescribe drugs”*
- We replace by the following sentence: *“This list of 38 explicit definitions necessitates additional confirmation by expert consensus before use in clinical practice.”*

**Response to Reviewer 3 Comments**

We thank Reviewer 3 for comments and constructive suggestions. We have carefully revised the manuscript according to each comment and suggestion. In the following, our responses are given in blue.

**Point 1.** The manuscript entitled 'Development of explicit definitions of potentially inappropriate prescriptions for antidiabetic drugs in patients with type 2 diabetes: a multidisciplinary qualitative study' provides some data about definitions of PIP in antidiabetics treatment by professionals, yet the expert opinions limited to certain region of the world may not be enough to provide a sounding definitive conclusion.

We thank the reviewer for this comment. We agree that expert opinion was limited to certain regions of the world. Such an approach has been used to develop most of the lists of potentially inappropriate prescriptions for older people. Yet, this approach did not limit the use of such lists to a single region of the world. For example, the Beers criteria were initially developed in a specific region of the USA, but have been used throughout the world. The same is true of the STOPP/START criteria (Ireland) or the FORTRA list (Germany), which are used far beyond their own countries. More importantly, the first lists of potentially inappropriate prescriptions for older people have attracted the attention of many researchers and clinicians, who have adapted the various lists to their local context.

As a result, we believe that the first movement towards explicit definitions of potentially inappropriate prescribing of antidiabetic drugs in patients with type 2 diabetes may lead to solid advances in research in this area.

**Point 2.** Line 51 states that 'prescription of antidiabetic drugs (ADs) can be challenging for non-specialist physicians'. This statement appear biased as prescription of antidiabetic drugs is generally challenging and needs involvement of different professionals particularly in the presence of comorbid conditions.

We thank the reviewer for this comment. We agree, the management of patients with type 2 diabetes should ideally involve different professionals. Prescribing diabetes treatments is therefore a global challenge for all healthcare professionals, including those who are not diabetes specialists or when management is carried out in an isolated, non-pluri-disciplinary context.

**In line with the reviewer’s comment, we proposed the following modifications in lines 60-62 (previous line 51) of the revised manuscript with track changes:** *“Managing diabetes is a challenge for all healthcare professionals, including those who are not diabetes specialists or when patients are managed in an isolated, non-multidisciplinary context.”*

**Point 3.** It would be advisable to correct grammatical errors throughout the document.
For e.g
Line 60 grammar error 'expert'' expert's
Line 64 grammar error 'to reduced' to reduce
Line 153 grammar error 'was validated' were validated

We thank reviewer for this comment. We had the manuscript proofread by a translator (David Fraser, Biotech Communication SARL, Ploudalmézeau, France) for the initial submission. We then added some new sentences after the first review, but we did not have enough time to perform English editing with a professional translator. This has been done for this revised version of our manuscript (David Fraser, Biotech Communication SARL, Ploudalmézeau, France).

The 11 approved modifications can be found in the following sections of the revised manuscript with track changes:

- **Lines 33-34:** *“A nominal group technique is a structured method that encourages all the participants to contribute and makes it easier to reach an agreement quickly”*
- **Lines 43-47:** *“The results of our qualitative study show that it is possible to develop a specific list of explicit definitions of potentially inappropriate prescriptions of antidiabetic drugs in patients with type 2 diabetes mellitus by gathering the opinions of healthcare professionals caring for these patients. This list of 38 explicit definitions necessitates additional confirmation by expert consensus before use in clinical practice”*
- **Lines 54-57:** *“Over the last decade, the management of patients with type 2 diabetes mellitus (T2DM) has undergone many changes; for example, a number of new antidiabetic drugs (ADs, including SGLT-2 inhibitors, GLP-1 receptor agonists, and DPP-4 inhibitors) have been approved as first-line treatments (either as monotherapies or in combination with other drugs). [1–5]. Managing diabetes is a challenge for all healthcare professionals, including those who are not diabetes specialists or when patients are managed in an isolated, non-multidisciplinary context.”*
- **Lines 66-69:** *“A professional consensus and clinical practice guidelines on the follow-up of patients with diabetes have been updated recently [6]. Patients with T2DM are mainly followed up by general practitioners [7–11]. However, various studies have shown that even after these recent updates, compliance with the guidelines is suboptimal – notably for patients followed up by general practitioners”*
- **Lines 75-95:** *“This approach is defined as implicit because it requires an expert’s assessment of the quality of care in relation to the patient’s condition and the medical literature [17, 18]. The Medication Appropriateness Index is currently the most widely used implicit method [18, 19]. However, implicit approaches have practical limitations because their application is time-consuming and requires specialized knowledge and skills.
  It is also possible to reduced PIPs by applying an explicit approach, i.e. predefined explicit criteria that combine the various items of information related to the prescription in the absence of an expert assessment (e.g. a prescription of metformin in a patient with an estimated glomerular filtration rate (eGFR) below 30 mL/min/1.73 m² is a PIP) [19, 20]. The value of explicit approaches for the detection PIPs has been well documented [21–24].
  Some experts recommend that implicit and explicit approaches should be combined for optimal patient management [24, 25].”*
- **Lines 110-113:** *“The nominal group technique has been widely used to gather the opinions of healthcare professionals and to generate new ideas [40–45].”*
- **Lines 119-121:** *“The present work involved a qualitative approach that had already been used to set up explicit definitions of PIP-ADs in hospitalized older patients”*
- **Lines 127-131:** *“These checklists relate to the sampling method, data collection setting, data collection method, respondent validation of findings, method of recording data, description of the derivation of themes, and inclusion of supporting quotations. The items are grouped into three domains: (i) research team and reflexivity, (ii) study design and (iii) data analysis and reporting.”*
- **Lines 141-145:** *“The inappropriateness of drug prescriptions can be evaluated in clinical pharmacology using two approaches: the first is based on a so-called “implicit” expert judgement, and the second uses explicit criteria. An implicit judgement is based on an expert’s evaluation of the quality of care with regard to the patient’s situation and guidelines on the use of drugs. In contrast, explicit criteria are based on predefined rules for the analysis of drug prescriptions, and do not require intervention by an expert.”*
- **Lines 152-155:** *“Explicit definitions cover situations considered by experts to be generally inappropriate, as defined in the literature or by expert consensus. However, when an explicit definition is applied to a given prescription, the absence of expert opinion means that the prescription’s inappropriateness cannot be confirmed. Therefore, explicit definitions correspond to PIPs.”*
- **Line 324-328:** *“Our qualitative study involved 30 healthcare professionals (10 diabetologists, 6 general practitioners, and 14 pharmacists) and generated 38 explicit definitions of PIP-ADs classified with regard to four contexts: (i) the potential need to temporarily discontinue a medication in the event of acute illness, (ii) the potential need to adjust the dosage regimen, (iii) inappropriate treatment initiation, and (iv) the need for additional monitoring of a patient with T2DM.*
